# NuclampFISH enables cell sorting based on nuclear RNA expression for chromatin analysis

**DOI:** 10.1186/s12864-025-11818-0

**Published:** 2025-07-01

**Authors:** Yifang Liu, Yuchen Qiu, Keqing Nian, Sara H. Rouhanifard

**Affiliations:** Department of Bioengineering, Northeastern University, Boston, MA USA

**Keywords:** SmFISH, RNA FISH amplification, ClampFISH, Transcription site, Transcriptional burst, Chromatin conformation

## Abstract

**Background:**

Transcriptional bursts are periods when RNA polymerase interacts with a DNA locus, leading to active gene transcription. This bursting activity can vary across individual cells, and analyzing the differences in transcription sites can help identify key drivers of gene expression for a specific target RNA. Scaffolding methods based on fluorescence in situ hybridization (FISH) have been widely used to amplify the fluorescent signal of RNAs and sort cells based on RNA expression levels. Examples include click-amplifying FISH (clampFISH) and hybridization chain reaction (HCR). However, these methods are limited in their ability to target and amplify transcription sites, due to the long probes’ hindered accessibility through cellular compartment membranes and crosslinked proteins. Thus, sorting based on transcriptional bursting has not been achieved. Additionally, the required formaldehyde fixation interferes with downstream analysis of chromatin and protein-binding interactions.

**Results:**

To address these challenges, we developed a platform, nuclear clampFISH (nuclampFISH) that integrates click-amplified FISH with reversible crosslinkers and allows access to the nucleus. We demonstrate that with optimized parameters and by eliminating the cytosol and cell membrane, this method enables the amplification of fluorescent signal for RNAs using a reversible crosslinker, enabling the sorting of cells based on nuclear RNA expression and is compatible with downstream biochemical analysis including chromatin conformation assays. We applied this assay to demonstrate that transcriptionally active cells have more accessible chromatin for a respective gene.

**Conclusions:**

This new method enables the sorting of cells based on transcriptional bursts. This method combines the specificity of a single-cell assay for detecting transcription sites with the throughput of flow cytometry to enable bulk assays such as chromatin conformation or other biochemical assays. Notably, the tools developed are highly accessible and do not require specialized computation or equipment.

**Supplementary Information:**

The online version contains supplementary material available at 10.1186/s12864-025-11818-0.

## Background

Transcription sites are specific loci where the RNA polymerase binds to the DNA to initiate the synthesis of RNA from a gene template. The presence of a transcription site by methods such as RNA FISH indicates that these genes are transcriptionally active or “bursting.” It is well established in the field that single cells have variable expression of transcription sites, indicating that they are in molecularly different states [1–4]. However, the specific mechanisms and regulatory machinery behind such variation are less understood. Transcription regulation, specifically bursting, involves the recruitment and release of RNA Pol II at the promoter. When this activity is very high, the expression of bursts can appear homogeneous, but when they are less frequent, it leads to cell-to-cell variability [5]. One of the major challenges is to understand the regulators of bursting.

Current methods for studying bursting primarily use RNA PolII chromatin immunoprecipitation coupled with sequencing (ChIP-seq) [6, 7]. This method informs on DNA–protein interactions and identifies actively transcribed regions. Other approaches enrich for nascent RNA prior to sequencing, enabling genome-wide profoling of newly synthesized transcripts [8]. However, these bulk techniques lack single-cell resolution. Single-molecule fluorescence in situ hybridization (smFISH) overcomes this by targetting introns to measure the frequency and duration of transcriptional bursts in individual, fixed cells [9, 10]. Live-cell imaging methods—such as MS2 stemloop tagging of nascent RNA—add realtime dynamics to this toolkit, allowing direct observation of transcriptional bursting in single cells [11]. Yet neither smFISH nor live imaging reports on RNA–protein interactions, and smFISH signals are too dim for flow cytometric separation based on transcriptional activity. An ideal method would combine gene specificity, single-cell and realtime sensitivity, bright signal for flow cytometry, and compatibility with downstream biochemical assays.

Various FISH-based scaffolding methods have been developed to enhance the fluorescent signals produced from RNA-binding oligonucleotide probes to enable high-throughput applications such as flow cytometry. These methods can all achieve high amplification [12–14] but lose signal integrity in nuclear RNA. One of these methods we developed is click-amplified FISH (clampFISH), which uses a “C” shaped probe to hybridize and form a double helix with the target RNA, followed by ligation using bioorthogonal click chemistry [15, 16]. The benefit of this method is that the ligated probes can survive stringent wash steps and achieve exponential amplification, and the backbone sequence can be modified for facile multiplexing. This method has also been recently expanded for faster analysis and higher throughput (clampFISH 2.0) [16].

Although HCR FISH can visualize nascent introns in nuclei, the linear amplification can produce weak nuclear-to-cytoplasmic contrast, particularly at the transcription site, and unreliable ON/OFF thresholding for flow sorting [17, 18]. To overcome these limitations, we established nuclampFISH to target nuclear RNA and transcription sites using a reversible crosslinker. We then used this amplification strategy to sort cells based on transcriptional activity for the first time and performed a chromatin accessibility assay to interrogate differences between transcriptionally active and inactive populations.

## Results

### ClampFISH and HCR probes are inaccessible to transcription sites

**Fig. 1:**
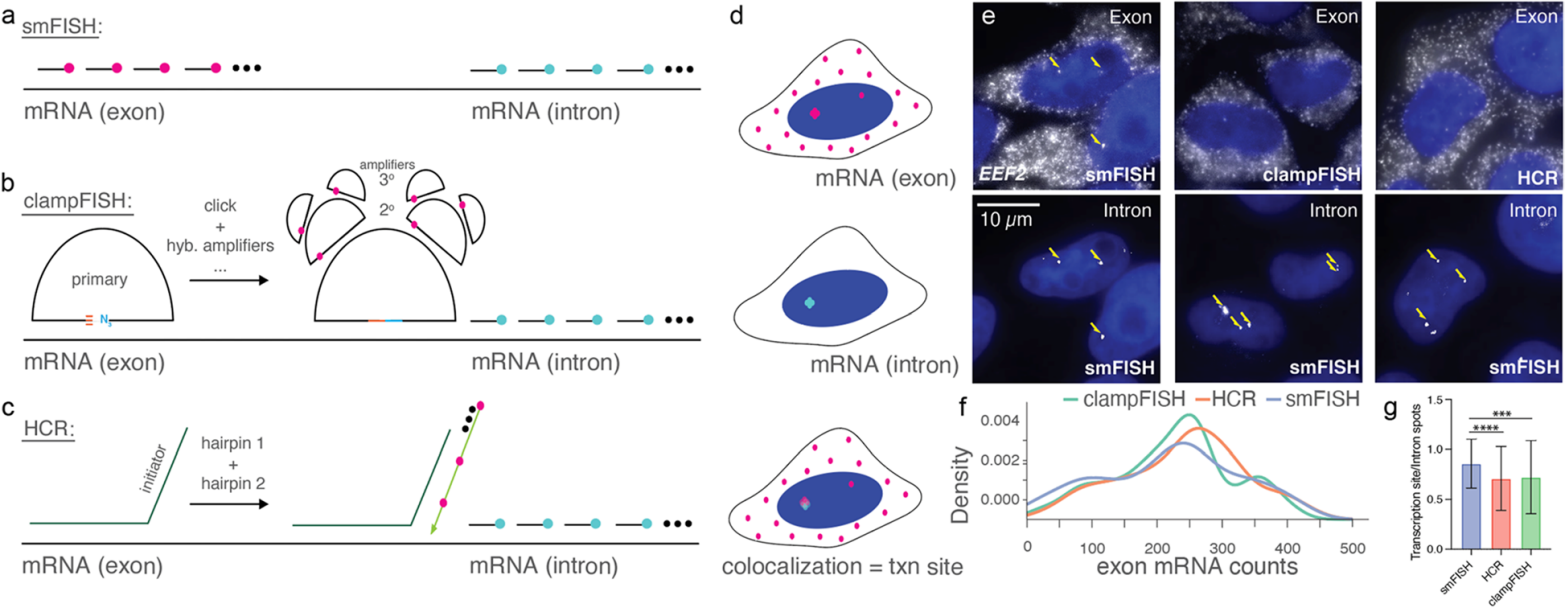
FISH-based methods to detect transcription sites include **a**. smFISH, **b**. clampFISH, and **c**. HCR FISH. **d** Overlaid FISH spots in mRNA exon and intron indicate transcription (txn) sites. **e** Images of EEF2 exons and smFISH images of EEF2 introns were used to show the colocalization and identify transcription sites. **f** Overlaid density plot of exon RNA count distribution for clampFISH (*n*=158 cells), HCR FISH (*n*=160 cells), and smFISH (*n*=157 cells) for two biological replicates. **g** quantification of transcription sites (txn sites) detected by method. A transcription site is defined as colocalization of exon probe with smFISH intron probe. Error bars represent the standard deviation from the mean. Statistical analysis was performed using a paired t-test. ***p < 0.0001, ****p < 0.00001

Previous reports have suggested that nuclear accessibility does not influence smFISH probes permeabilization (Fig. 1a), but it presents a challenge for FISH-based amplification, such as clampFISH and hybridization chain reaction (HCR) (Fig. 1b, c) [15]. Transcription sites can be detectable using these methods, but sufficient amplification and specificity for high-throughput applications such as flow sorting has remained a challenge. Transcription sites reside in the nucleus and may be detected by the colocalization of exon and intron FISH probes for the same transcript (Fig. 1d). To assess the labeling efficiency of molecular scaffold-based amplification strategies for detecting transcription sites, we labeled *EEF2* mRNA with smFISH, clampFISH, and HCR (Fig. 1e). This target has an average of 1.5 transcription sites in most HeLa cells, as detected by the colocalization of smFISH intron and exon probes in the nucleus (Fig. 1e). ClampFISH and HCR probes were designed to target the *EEF2* exon sequence. They were counterstained by the smFISH probes that targeted the *EEF2* intron. We found that clampFISH and HCR can reliably detect exon mRNA in cytoplasm, but the signal from the transcription site was lost or not specific. The nuclear exon spots overlaid well with nuclear intron spots (arrow), indicating that smFISH can detect transcription sites specifically (Fig. 1e). The average signal to noise ratios for HCR, clampFISH and smFISH were 3.27 *+ 1.74*,* 1.35 + 0.28*,* and 1.43 + 0.26 respectively.* The distribution of exon FISH spots in clampFISH, HCR, and smFISH overlaps and the median number of the RNA spots counts in these three conditions are consistent: HCR: 250 *±* 4.2, clampFISH: 236 *±* 8.5, smFISH: 235 *±* 7.4 (Fig. 1f). However, quantification of transcription sites per cell shows significantly fewer transcription sites per intron signal detected by clampFISH (*p* = 0.0006) and HCR (*p* = 0.0001) for the same transcript (Fig. 1g) in addition to the transcription sites being substantially smaller than they appear by smFISH (Fig. 1e).

### Nuclear isolation and method optimization enable exponential amplification of nuclear RNA

**Fig. 2:**
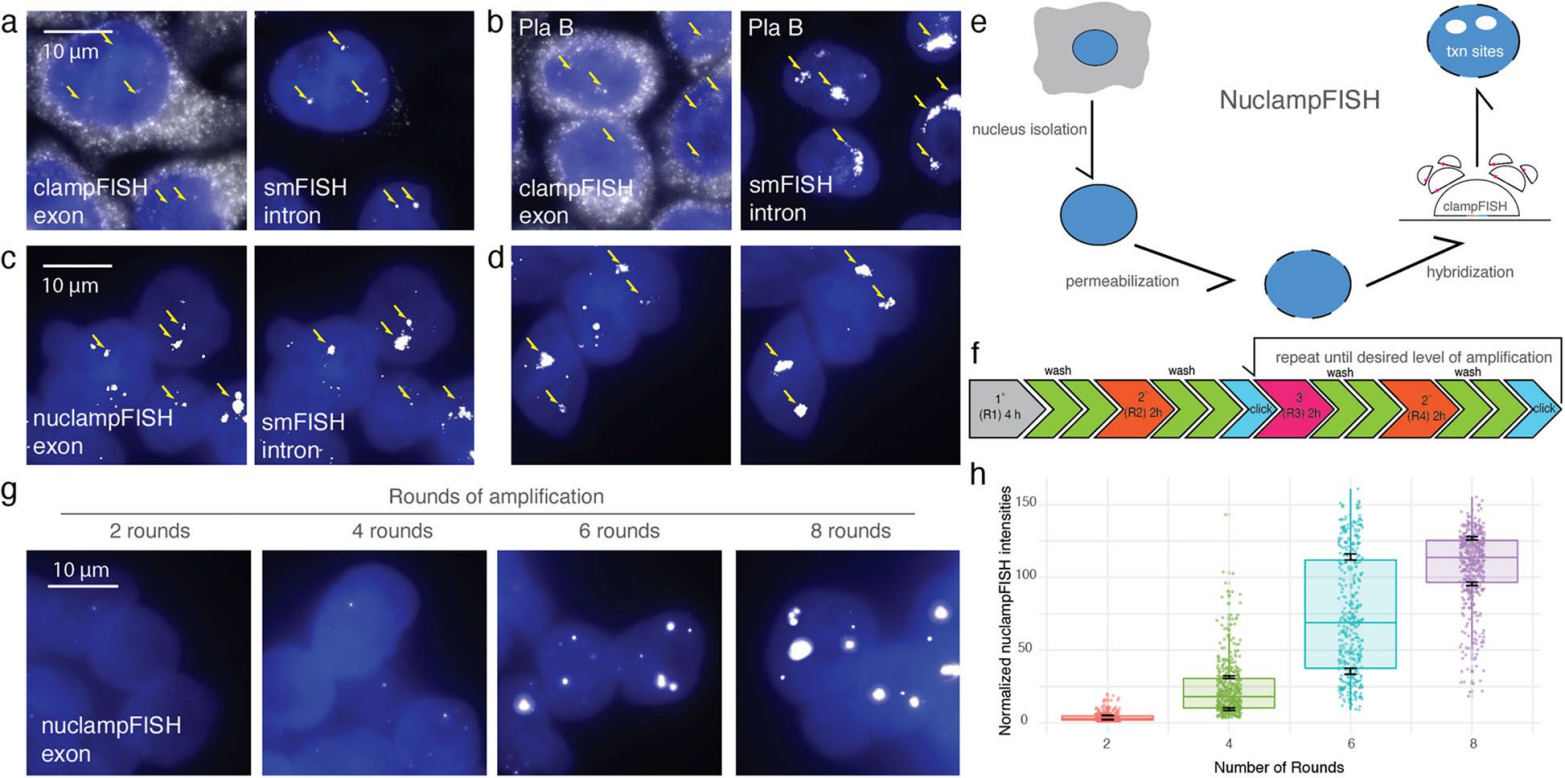
Method development and workflow of nuclampFISH. **a** clampFISH images after 2 rounds of amplification of EEF2 exon and smFISH images of EEF2 intron without Pla B treatment. The arrow indicates the transcription sites. **b** clampFISH images of EEF2 exon and smFISH images of EEF2 intron after Pla B treatment. **c** NuclampFISH images of EEF2 exon after nucleus isolation and smFISH images of EEF2 intron. **d** NuclampFISH images of EEF2 exon after nucleus isolation and modified hybridization parameters, and smFISH images of EEF2 intron. **e** Workflow of nuclampFISH methods. **f** Timeline of nuclampFISH process. **g** Amplification efficiency of nuclampFISH, two rounds, four rounds, six rounds, eight rounds of nuclampFISH images were shown. **h** Box and whisker plot of nuclampFISH spots intensities in each round of amplification. *n* = 3 biological replicates

One hypothesis is that FISH amplification strategies are not capable of probing transcription sites because the probe binding is inefficient, and a larger transcriptional burst would not have this problem. To test whether increasing the number of nascent transcripts for our target transcript would enhance the signals to a detectable level, we treated cells with Pladienolide B (Pla B) [19]. This splicing inhibitor induces the transcription of *EEF2* by blocking the assembling process of spliceosome [20]. We applied clampFISH probes targeting *EEF2* exon using the original clampFISH protocol [15] with and without the presence of Pla B. We found that even after Pla B treatment, clampFISH still exhibited a dim signal even though the smFISH signal had a 1.73-fold increase as without Pla B treatment (Fig. 2a, b, Supplementary Fig. 1). The relatively large smFISH foci (> 2 μm) observed after nuclei isolation reflect both the selection of a gene with exceptionally large introns and robust transcriptional bursts [21], as well as the effects of our isolation and PLA treatment, which enhance probe accessibility and inhibit splicing, thereby amplifying and stabilizing the transcriptional signal.

We next hypothesized that the molecular scaffold size of the clampFISH probe prevents itself from diffusing through the cytoplasm to access the target in the nucleus. To test this hypothesis, we removed the cell membrane and cytoplasm, then applied the clampFISH probes targeting *EEF2* exons with two rounds of amplification (i.e., primary probe and secondary probe). After nuclear isolation, the signal intensities of transcription sites inside the cell nucleus and were improved significantly (Fig. 2c, Supplementary Fig. 2). Next, we optimized the inclusion of surfactant and salt to improve probe binding. Triton X-100 enhances cell permeability, and salt drives probe hybridization with the target mRNA. After adding Triton X-100, we found that the nucleus clampFISH signal intensities increased (Fig. 2d, Supplementary Fig. 2). These values further increased by increasing the salt concentration from 2X SSC to 5X SSC (Fig. 2d, Supplementary Fig. 2). The same result was observed for another nuclear RNA target, *NEAT1*, a long non-coding RNA (Supplementary Fig. 3) and *TMSF1* exons (Supplementary Fig. 4).

Next, we optimized a method of probe delivery that would specifically and exponentially amplify the nuclear signal. We used the same primary probes used for the *EEF2* exon and amplified the signal using fluorescent secondary and tertiary probes with a click reaction performed (Fig. 2f). We performed the nuclampFISH protocol with varying stopping points: two rounds (primary and secondary), four rounds (primary, secondary, tertiary, and secondary again), six rounds, and eight rounds. We observed bright, amplified spots with increasing fluorescent signal as amplification rounds increased (Fig. 2g, h). Furthermore, we observed an exponential rate of amplification,1.74-fold per round, indicating that the amplifiers’ binding efficiency is 87.1% of the theoretical doubling intensity per round. This rate did not slow down even when we reached eight rounds of amplification, indicating that further amplification could be achievable.

### NuclampFISH is compatible with intron targeting and reversible crosslinking

**Fig. 3:**
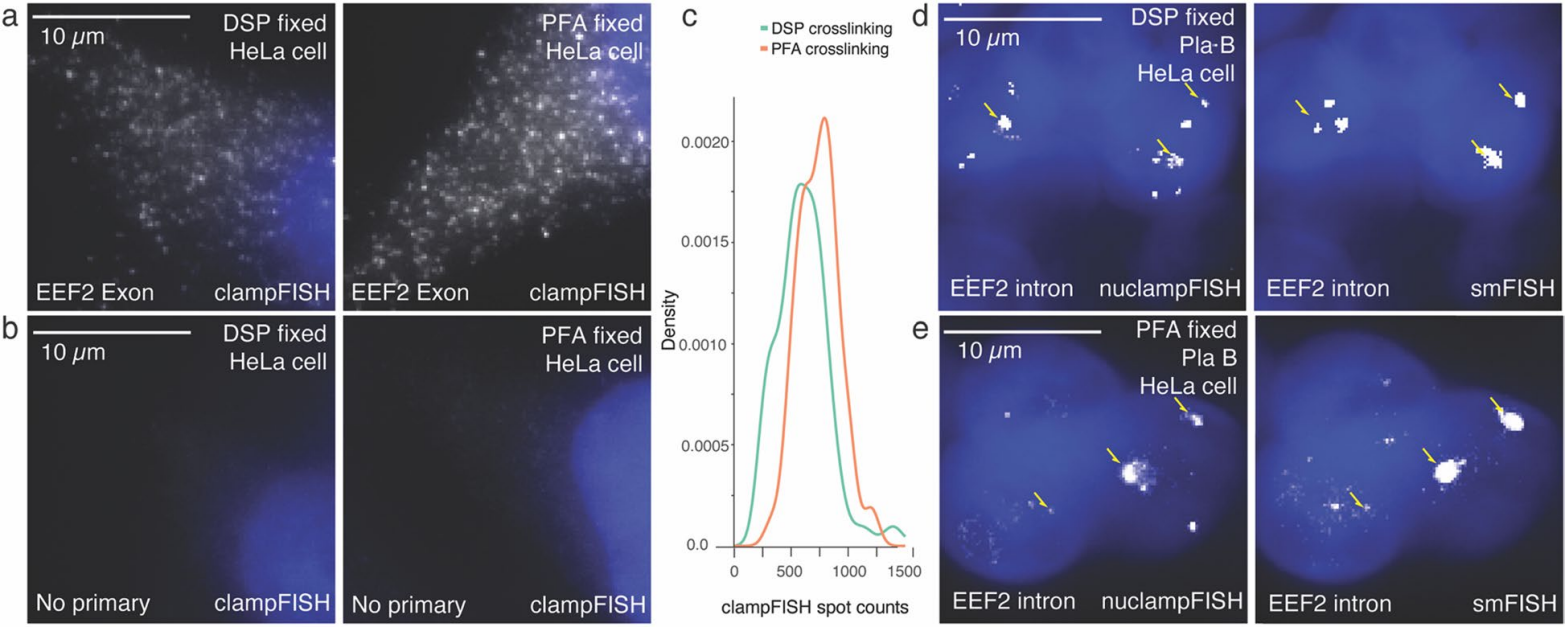
Application of reversible crosslinker to nuclampFISH. **a** HeLa cells stained for EEF2 exon mRNA using clampFISH after DSP (left) or formaldehyde (right) crosslinking. **b** HeLa cells processed with clampFISH secondary probes only, following DSP (left) or formaldehyde (right) crosslinking. **c** Density plot showing percell EEF2 mRNA counts in DSP (*n* = 100 cells) versus formaldehydetreated (*n* = 100 cells) samples. **d** NuclampFISH (left) and smFISH (right) images of EEF2 intron in isolated nuclei following Pladienolide B treatment under DSP crosslinking. **e** NuclampFISH (left) and smFISH (right) images of EEF2 intron in isolated nuclei following Pladienolide B treatment under formaldehyde crosslinking

FISH-based methods require fixation and permeabilization before probe hybridization, including those with molecular scaffolding for fluorescence amplification. Formaldehyde crosslinking is conventionally used for FISH-based assays [22, 23]. However, it can interfere with downstream analysis, such as mass spectrometry, biochemical assays, and chromatin profiling [24, 25]. To overcome the challenge of formaldehyde, we tested the compatibility of several alternative crosslinkers with smFISH and clampFISH, including glutaraldehyde, methanol, disuccinimidyl sulfoxide (DSSO), and dithiobis (succinimidyl propionate) (DSP). We found that DSSO and DSP have comparable performance with formaldehyde crosslinking (Supplementary Fig. 5). We chose DSP crosslinking because this is chemically reversible, and DSSO is reversible by mass spectrometry. DSP could be applied to a broader variety of downstream applications. DSP is a crosslinker that can be easily reversed by 10–50 mM dithiothreitol (DTT) or tris(2-carboxyethyl)phosphine(TCEP) at pH 8.5 [26]. We compared the performance of formaldehyde and DSP fixation by targeting the *EEF2* mRNAs using clampFISH exon probes and without exon probes as negative control in whole cells (Fig. 3a, b). We found that the RNA count distribution of DSP crosslinking is comparable to formaldehyde crosslinking with a mean fluorescence intensity (MFI) of 615.16 *±* 248.65 and 740.27 *±* 178.9 respectively (Fig. 3c). We applied the nuclampFISH method to target transcription sites using DSP crosslinking and formaldehyde crosslinking with probes complementary to the *EEF2* intron mRNA sequence. We observed that the nuclampFISH spots colocalized with smFISH spots at transcription sites, indicating that the protocol is compatible with DSP for targeting nuclear RNA while maintaining specificity (Fig. 3d, e).

### NuclampFISH enables cell sorting based on nuclear RNA expression

**Fig. 4:**
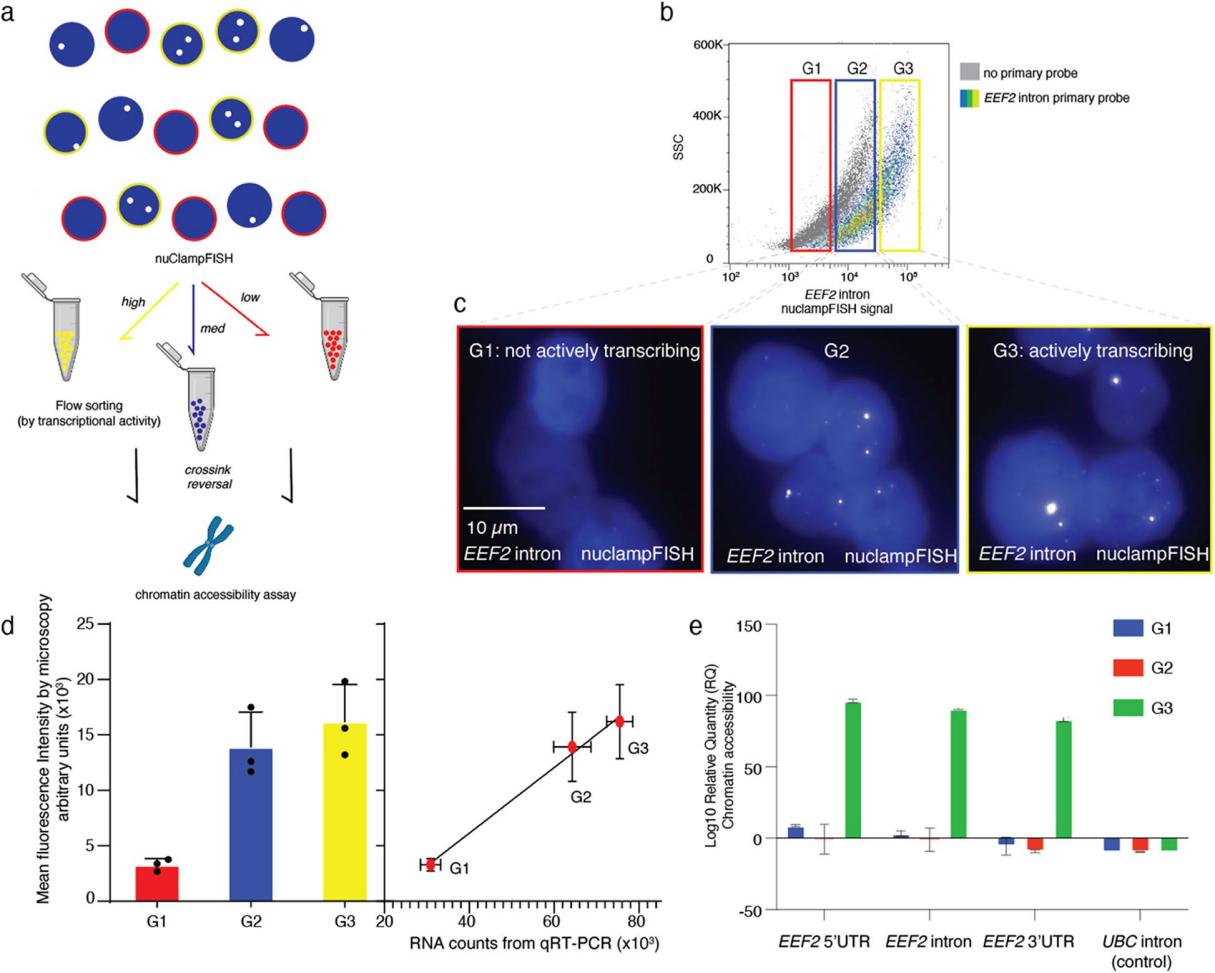
NuclampFISH workflow, sorting, and chromatin accessibility analysis. **a** Schematic of the nuclampFISH protocol, fluorescence-activated nuclear sorting, and downstream chromatin accessibility assay. **b** Pseudocolor flow‐cytometry plot of EEF2 intron fluorescence in HeLa nuclei. Gates for low (G1), medium (G2), and high (G3) expression define sorting populations. **c** Representative fluorescence micrographs of sorted G1, G2, and G3 nuclei. **d** Left: Bar graph of mean nuclampFISH signal intensity in each sorted population. Right: Correlation between qRTPCR–measured intron abundance and nuclampFISH fluorescence intensity; equal numbers of nuclei were analyzed per group. **e** Chromatin accessibility quantified by log₁₀ relative quantity (RQ) from 5 × 10⁵ sorted nuclei per group, indicating open‐chromatin levels for EEF2 intron regions in G1, G2, and G3

One primary application is that nuclampFISH enables sorting cells based on nuclear RNA expression by detecting the active transcription sites of a single mRNA. We used nuclampFISH to sort cells based on the expression of actively transcribing *EEF2* (i.e., clampFISH primary probes targeting the *EEF2* intron). Compared to the negative control, which included each round of amplifier probe, the positive group, which was treated with four rounds of amplification, showed almost a complete decade shift in fluorescence by flow cytometry detection (Fig. 4b). We set three gates (G1, G2, G3 with increased nuclampFISH signal) to sort cells based on the expression level of transcription sites (Fig. 4b). After collecting the cells from each gate, we imaged them using fluorescence microscopy (Fig. 4c). We found that the nuclampFISH signal on transcription sites, as shown by microscopy, corresponded to the increasing fluorescent intensity observed by flow cytometry (Fig. 4c, d, Supplementary Fig. 6). We extracted the RNA from the sorted cells and conducted RT-qPCR to quantify the levels of *EEF2* intron from each gated population. The RNA counts correlated significantly with nuclampFISH pixel intensities acquired by microscopy after sorting (*r* = 0.93; Fig. 4d) and nuclampFISH spots quantity (Supplementary Fig. 6).

### NuclampFISH enables cell sorting and crosslink reversal for chromatin accessibility analysis

Chromatin accessibility assays such as DNA sequencing-based methods facilitate characterizing active regulatory elements during active transcription [27, 28]. However, these are typically bulk assays that combine heterogeneous cell populations for analysis rather than single-cell assays. Single-cell assays such as smFISH can analyze gene-specific, nascent RNA; however, these assays do not inform DNA accessibility. We sought to combine the single-cell and gene-specific benefits of FISH-based assays with the sensitivity of bulk assays to detect chromatin structure and explore chromatin conformation during active transcription of a given gene.

As a critical first step, we hypothesized that the chromatin accessibility assay would be compatible with DSP-crosslinked cells for which the crosslinking was reversed. Chromatin accessibility assays report on the “openness” of chromatin based on the effectiveness of qPCR in a region of interest. Crosslinking the nucleic acids and proteins surrounding the region of interest can lead to misinterpretation of the results as closed when it could be a crosslinking artifact. As a proof-of-concept, we crosslinked the cells with DSP and compared the *EEF2* chromatin accessibility of the DSP reversal group with the no DSP reversal group; the reversal group exhibited a 3.16-fold increase in signal (Supplementary Fig. 7), indicating that crosslink reversal is compatible with the chromatin accessibility assay.

Next, we DSP crosslinked HeLa cells, labeled *EEF2* introns using nuclampFISH with four rounds of amplification, and collected the sorted nuclei from the G1, G2 and G3 groups. After reversing the crosslinking, we performed chromatin accessibility analysis at the genomic region harboring the *EEF2* intron as well as the 3’ and 5’ regions of the same transcript. We observed that the chromatin accessibility of the G3 group (i.e., the group with the highest level of intron signal for *EEF2* mRNA) significantly increased compared to the G1 group (i.e., the group with limited “burstiness”; *p* < 0.0001; Fig. 4e). As a control, we analyzed the housekeeping gene *UBC*, which has comparable expression levels to EEF2 (916.7 nTPM vs. 1069.7 nTPM, respectively, based on the GTEx protein atlas), and found no significant differences in chromatin accessibility across the G1, G2, and G3 groups (Fig. 4e). These results suggest that active transcription correlates with increased chromatin accessibility at the gene of interest, independent of baseline expression level.

## Discussion

Here, we established nuclampFISH, an amplified FISH method for targeting nuclear RNA and transcription sites. This method achieves the goal of specific detection of nuclear RNA (including transcription sites) and amplifying the FISH signal of nuclear RNA (including transcription sites). Based on the specific and amplified nuclampFISH signal, we offer a platform to separate cells according to the expression level of transcription sites for downstream analysis to understand transcription better. We combine the FISH detection and chromatin analysis with a chemical reversible crosslinker DSP.

We demonstrated that, compared to clampFISH and HCR FISH at transcription sites, nuclampFISH achieves amplification and highly specific detection while retaining the exponential amplification kinetics of clampFISH. Although all three methods can detect abundant nuclear RNAs—such as the lncRNA NEAT1 [15]—the total fluorescence for nuclear targets falls short of the increases seen in the cytoplasm, indicating reduced labeling efficiency in the nucleus. Indeed, similar to the impeded nuclear access reported for long DNA FISH probes [29], the bulky, clickligated design of clampFISH probes faces steric hindrance within densely packed nascent RNP complexes at transcription loci, leading to poor hybridization efficiency, elevated background, and an inability to accumulate sufficient nuclear signal. NuclampFISH overcomes these limitations by using probe designs and conditions optimized for nuclear penetration, yielding stronger, more reliable detection of transcription-site RNA without sacrificing amplification capacity.

We sorted nuclei based on nuclampFISH-measured transcriptional burst intensity to enrich for distinct ON and OFF states. In polyploid HeLa cells, more than two active loci per nucleus are expected, and the exponential amplification of nuclampFISH precludes direct counting of individual transcripts. As a result, nuclear foci likely consist of both genuine transcription‐site clusters and unspliced introns, each reflecting current or recent promoter activity. Although a small subset of sorted nuclei may lack obvious bursts—likely due to nonspecific carryover during nuclear isolation—the robust enrichment of intronic signal by RT-qPCR and our imaging analysis confirm that the sorting strategy reliably captures populations with differential transcriptional activity.

While nuclampFISH enables sorting of nuclei based on total fluorescence intensity, it does not resolve underlying differences in transcription site architecture—for example, whether high fluorescence reflects a single large transcriptional burst or multiple smaller ones. Because sorting is based on integrated signal, such distinctions are collapsed into the same bin. This limitation should be considered when interpreting downstream measurements, such as microscopy or RT-qPCR, as the sorted populations may include heterogeneous transcriptional states. Interestingly, while transcriptional activity increases progressively from G1 to G3, chromatin accessibility does not show a proportional stepwise change—suggesting that for highly expressed loci like *EEF2*, transcriptional upregulation may occur through post-accessibility mechanisms such as increased polymerase recruitment or burst frequency rather than further chromatin remodeling.

Unlike epiMERFISH [30] and seqFISH [31], which combine epigenetic profiling and transcriptional readout while retaining singlecell spatial positions, our nuclampFISH workflow deliberately sacrifices spatial context in favor of functional enrichment. By sorting nuclei on the basis of transcriptional “burst” activity, we selectively isolate populations in defined ON or OFF states, thereby enabling downstream biochemical and omics assays—such as proteomics, chromatin immunoprecipitation, or ATACseq—that would be difficult or impossible at singlecell resolution in situ. This populationlevel strategy thus complements spatially resolved methods by providing a means to biochemically characterize transcriptionally homogeneous cohorts. Although our chromatin accessibility data provide a proof of principle for downstream biochemical analyses, future studies will include Pol II ChIP–qPCR across G1, G2, and G3 to rigorously quantify factor enrichment after the nuclampFISH procedure.

## Conclusions

In summary, we report developing a method for the specific detection of nuclear RNA and transcription sites. This assay broadens the application of clampFISH for transcription sites by amplifying specific FISH signals, thus enabling the separation of the cells for downstream analysis, including chromatin analysis, proteomics, and transcriptional profiling.

## Methods

### Tissue culture conditions

HeLa cells were handled according to the manufacturer’s protocol and maintained in high-glucose DMEM with GlutaMax (FisherSci cat# 10566016), 10% FBS, and 1% Pen-Strep (FisherSci cat# BW17-602E). Cell lines tested PCR-negative for mycoplasma contamination (ATCC cat# 30–1012 K).

### Drug treatment for splicing Inhibition

Cells were treated with 1 µM Pladienolide B (Tocris Biosciences #6070500U) for 4 h to achieve splicing inhibition as described by previous work [32].

### SmFISH

The smFISH detection was performed as previously described [23]. Sequences for smFISH probes can be found in Supplementary Table 1.

### HCR FISH

HCR FISH was conducted using the HCR™ RNA-FISH Bundle from Molecular Instruments. We followed the manufacturer’s protocol [12, 33].

### NuclampFISH protocol

#### Nucleus extraction

Minute™ Single Nucleus Isolation Kit for Tissues/Cells (Invent Biotechnologies cat# SN-047) was used to extract nuclei from whole cells. 5 × 10 ^6^- 10 ^7^ cells were collected and washed by pre-cold 1X PBS once. 200 µL lysis buffer was added to the tube, and the cells were ground with the pestle provided 20–30 times. Another 400 µL lysis buffer was added to the tube and incubated at 4 °C for 10 min. After incubation, all cell lysate was transferred into a filter in a collection tube. The nuclei pellet was obtained by centrifuge at 600 X g for 5 min. The supernatant was removed carefully and discarded. The pellet was resuspended in 0.8 mL cold washing buffer by pipetting up and down 20–30 times. The washed nuclei were centrifuged at 500 X g for 5 min. The supernatant was removed and discarded. The nuclei were ready to proceed into the fixation step.

#### Formaldehyde and DSP fixation

After cell nuclei were isolated and washed, 3.7% formaldehyde (1X PBS and diluted in Nuclease-Free water) and 500 µM DSP (ThermoFisher #PIA35393, 1 mg stock dissolved in 50 µL DMSO and then diluted in 1X PBS) were directly added to the pellet to fix nuclei, the incubation time was 10 min. For 5 × 10 ^6^- 10 ^7^ cells, 5 mL 3.7% formaldehyde and DSP were added. After fixation, the nuclei were incubated with 1X PBS/0.1%triton for 5 min twice to improve permeabilization. After this, the nuclei were incubated with clampFISH wash buffer/0.25% triton for 20 min to achieve further permeabilization. The nuclei were ready to do clampFISH treatment.

#### ClampFISH

The clampFISH procedure was conducted as in previous work [15] with several modifications to increase nuclear RNA FISH signal.

We cultured cells on glass coverslips until they reached approximately 70% confluence. The cells were washed twice with PBS and then fixed in 4% formaldehyde in PBS at room temperature for 10 min. After aspirating the formaldehyde, we rinsed the cells twice with PBS and stored them in 70% ethanol at 4 °C. For hybridization, we incubated the cells for at least 4 h at 37 °C in a buffer containing 10% dextran sulfate, 5× SSC, 0.25% Triton-X-100 and 20% formamide, along with 0.5 µL of the primary ClampFISH probe. Probes were designed according to Rouhanifard et al. [15].

Next, we performed two 30-minute washes at 37 °C in wash buffer (2× SSC, 10% formamide). We then incubated the cells for at least 2 h at 37 °C in a hybridization buffer with 1 µL of the secondary ClampFISH probe, followed by the same wash procedure. After the second wash, we initiated the click chemistry reaction by adding a solution of 75 µM CuSO4 premixed with 150 µM BTTAA ligand (Jena Biosciences) and 2.5 mM freshly prepared sodium ascorbate (Sigma) in 2× SSC. The samples were incubated for 30 min at 37 °C and briefly rinsed with wash buffer.

We continued the amplification cycles by alternating between secondary and tertiary ClampFISH probes, followed by the respective wash steps until the desired signal amplification was achieved. After the final wash, the cells were rinsed once with 2× SSC containing DAPI and once with antifade buffer (10 mM Tris, pH 8.0, 2× SSC, 1% w/v glucose). The samples were then mounted for imaging in an antifade buffer containing catalase (Sigma) and glucose oxidase (Sigma) to prevent photobleaching.

For suspension cells, 0.25% Triton X-100 was added to the wash buffer; the cells were stored in 2X SSC/0.25% triton after clampFISH with desired rounds ready for flow cytometry sorting. Sequences for clampFISH probes can be found in Supplementary Table 1.

### Flow cytometry and sorting

For nuclampFISH, we sorted on *the EEF2* intron and *NEAT1* based on the nuclampFISH signal using the BECKMAN Sorter (Cell Sorter), which uses 638 nm excitation and 660 nm emission.

### DSP cross-linking reversal

To reverse the DSP crosslinking for chromatin accessibility assays, the sorted cells were treated with 25 mM DTT at 37 °C for 30 min, according to the manufacturer’s instructions. The cells were then washed with 1X PBS once and ready for the chromatin accessibility assay.

### RNA extraction, RT-qPCR

RNA was extracted using Trizol (Thermo Fisher #15596026) and resuspended in NF water. DNase (Thermo Fisher #AM1907) was used to remove the contaminant DNA. RNA was reverse transcribed (Fisher Scientific #18–080-044). The Luna qPCR (NEB #M3004) was used to quantify the generated cDNA. The OneTaq RT-PCR mix (NEB #E5315) was used to amplify the generated cDNA. Sequences for primers can be found in Supplementary Table 1.

### Chromatin accessibility assay

After nuclei were sorted out based on the nuclampFISH signal and DSP crosslinking was cleaved, the cell’s chromatin was extracted, and the chromatin accessibility assay (EpiQuik Chromatin Accessibility Assay Kit) was performed according to the manufacture protocol (Epigentek, #P-1047-48) with one modification. The manufacturer recommends 2uL of nuclease in 50uL total but this over-digested the chromatin from the nucleus isolated samples. We optimized the concentration to 0.2ul of nuclease in 50 ul total volume. Chromatin accessibility was quantified with the EpiQuik Chromatin Accessibility Assay Kit (Epigentek, P-1047-48) according to the manufacturer’s instructions. Fold enrichment (FE) values for the target gene EEF2 were normalised to the housekeeping gene UBC: an internal reference (U_mean) was obtained by averaging FE values from three UBC amplicons (5′ UTR, intron, and 3′ UTR). Relative accessibility for each EEF2 region was calculated as RQ = 2^{-(FE_target − U_mean)} and log-transformed (log₁₀[RQ + 10^{−10}]) before statistical analysis and plotting.

### Image acquisition

Microscopy was performed using a Nikon inverted research microscope eclipse Ti2-E/Ti2-E/B using a Plan Apo λ 20X/0.75 objective or Plan Apo λ 100X/1.45 oil objective. The Epi-fi LED illuminator linked to the microscope assured illumination and controlled the respective brightness of four types of LEDs of different wavelengths. Images were acquired using the Neutral Density (ND16) filter for probes coupled with Alexa 488, Alexa 594, Alexa 647, and cy3. Images were acquired and processed using ImageJ. Images acquired using the Neutral Density (ND16) filter are false-colored gray.

### Image analysis and quantification

We segmented and thresholded the images using a custom Matlab software suite [5]. Transcription sites were identified by bright nuclear intron spots. Cell segmentation was performed manually by drawing boundaries around non-overlapping cells. The software then fits each detected spot to a two-dimensional Gaussian profile, specifically on the z plane on which it occurs, to ascertain subpixel-resolution spot locations. Colocalization took place in two stages. In the first stage, the software searched for the nearest neighbor for the smFISH probe with the nuclampFISH probe within a 2.5-pixel (360-nm) window to determine colocalization. All fluorescence images were acquired using identical microscope settings—including exposure time, gain, and illumination intensity—to ensure quantitative comparability across experimental groups. Raw image stacks were maximumintensity projected in ImageJ, and the resulting projections for each channel were contrastadjusted using the same window and level parameters. No nonlinear transformations (e.g., gamma correction) were applied. Processed images presented in figures thus reflect true relative differences in signal intensity and spatial distribution, allowing direct visual comparison between conditions. For illustrative single-cell analysis (e.g., Supplementary Fig. 6B), we manually selected a representative set of 20 cells from a single replicate to highlight the distribution across transcriptional gates. For quantitative comparisons (e.g., Fig. 4), we analyzed the full population of segmented cells from multiple biological replicates (typically > 200 cells per condition). The analysis software computes the mean intensity value for each replicate. These replicate-level means were used for statistical comparison across conditions using a paired t-test. Error bars represent standard error of the mean (SEM) across replicates.

## Supplementary Information

Supplementary Material 1.

Supplementary Material 2.

## Data Availability

All data generated or analyzed during this study are included in this published article [and its supplementary information files].

## References

[1] Raj A, Peskin CS, Tranchina D, Vargas DY, Tyagi S. Stochastic mRNA synthesis in mammalian cells. PLoS Biol. 2006;4:e309. 17048983 10.1371/journal.pbio.0040309 PMC1563489

[2] Zenklusen D, Larson DR, Singer RH. Single-RNA counting reveals alternative modes of gene expression in yeast. Nat Struct Mol Biol. 2008;15:1263–71. 19011635 10.1038/nsmb.1514 PMC3154325

[3] Suter DM, Molina N, Gatfield D, Schneider K, Schibler U, Naef F. Mammalian genes are transcribed with widely different bursting kinetics. Science. 2011;332:472–4. 21415320 10.1126/science.1198817

[4] Coleman RA, Liu Z, Darzacq X, Tjian R, Singer RH, Lionnet T. Imaging transcription: past, present, and future. Cold Spring Harb Symp Quant Biol. 2015;80:1–8. 26763984 10.1101/sqb.2015.80.027201 PMC4915995

[5] Raj A, Rifkin SA, Andersen E, van Oudenaarden A. Variability in gene expression underlies incomplete penetrance. Nature. 2010;463:913–8. 20164922 10.1038/nature08781 PMC2836165

[6] Park PJ. ChIP-seq: advantages and challenges of a maturing technology. Nat Rev Genet. 2009;10:669–80. 19736561 10.1038/nrg2641 PMC3191340

[7] Johnson DS, Mortazavi A, Myers RM, Wold B. Genome-wide mapping of in vivo protein-DNA interactions. Science. 2007;316:1497–502. 17540862 10.1126/science.1141319

[8] Wissink EM, Vihervaara A, Tippens ND, Lis JT. Nascent RNA analyses: tracking transcription and its regulation. Nat Rev Genet. 2019;20:705–23. 31399713 10.1038/s41576-019-0159-6 PMC6858503

[9] Wheat JC, Sella Y, Willcockson M, Skoultchi AI, Bergman A, Singer RH, et al. Single-molecule imaging of transcription dynamics in somatic stem cells. Nature. 2020;583:431–6. 32581360 10.1038/s41586-020-2432-4 PMC8577313

[10] Senecal A, Munsky B, Proux F, Ly N, Braye FE, Zimmer C, et al. Transcription factors modulate c-Fos transcriptional bursts. Cell Rep. 2014;8:75–83. 24981864 10.1016/j.celrep.2014.05.053 PMC5555219

[11] Larson DR, Zenklusen D, Wu B, Chao JA, Singer RH. Real-time observation of transcription initiation and elongation on an endogenous yeast gene. Science. 2011;332:475–8. 21512033 10.1126/science.1202142 PMC3152976

[12] Choi HMT, Schwarzkopf M, Fornace ME, Acharya A, Artavanis G, Stegmaier J, et al. Third-generation in situ hybridization chain reaction: multiplexed, quantitative, sensitive, versatile, robust. Development. 2018;145. 10.1242/dev.165753 PMC6031405 29945988

[13] Garcia-Perez L, van Eggermond MCJA, Maietta E, van der Hoorn M-LP, Pike-Overzet K, Staal FJT. A novel branched DNA-Based flowcytometric method for Single-Cell characterization of gene therapy products and expression of therapeutic genes. Front Immunol. 2020;11:607991. 33584681 10.3389/fimmu.2020.607991 PMC7876092

[14] Kishi JY, Lapan SW, Beliveau BJ, West ER, Zhu A, Sasaki HM, et al. SABER amplifies FISH: enhanced multiplexed imaging of RNA and DNA in cells and tissues. Nat Methods. 2019;16:533–44. 31110282 10.1038/s41592-019-0404-0 PMC6544483

[15] Rouhanifard SH, Mellis IA, Dunagin M, Bayatpour S, Jiang CL, Dardani I, et al. ClampFISH detects individual nucleic acid molecules using click chemistry–based amplification. Nat Biotechnol. 2018;37:84–9. 10.1038/nbt.4286 PMC6511493 30418432

[16] Dardani I, Emert BL, Goyal Y, Jiang CL, Kaur A, Lee J, et al. ClampFISH 2.0 enables rapid, scalable amplified RNA detection in situ. Nat Methods. 2022;19:1403–10. 36280724 10.1038/s41592-022-01653-6 PMC9838136

[17] Yuan Y, Chen Q, Brovkina M, Clowney EJ, Yadlapalli S. Clock-dependent chromatin accessibility rhythms regulate circadian transcription. PLoS Genet. 2024;20:e1011278. 38805552 10.1371/journal.pgen.1011278 PMC11161047

[18] Hoffman JA, Trotter KW, Day CR, Ward JM, Inoue K, Rodriguez J, et al. Multimodal regulatory elements within a hormone-specific super enhancer control a heterogeneous transcriptional response. Mol Cell. 2022;82:803–e8155. 35077705 10.1016/j.molcel.2021.12.035 PMC8897972

[19] Post-transcriptional splicing. can occur in a slow-moving zone around the gene. https://elifesciences.org/reviewed-preprints/91357. Accessed 13 Dec 2023. 10.7554/eLife.91357 PMC10997330 38577979

[20] Kotake Y, Sagane K, Owa T, Mimori-Kiyosue Y, Shimizu H, Uesugi M, et al. Splicing factor SF3b as a target of the antitumor natural product pladienolide. Nat Chem Biol. 2007;3:570–5. 17643112 10.1038/nchembio.2007.16

[21] Coté A, O’Farrell A, Dardani I, Dunagin M, Coté C, Wan Y, et al. Post-transcriptional splicing can occur in a slow-moving zone around the gene. Elife. 2024;12. 10.7554/eLife.91357 PMC10997330 38577979

[22] Femino AM, Fay FS, Fogarty K, Singer RH. Visualization of single RNA transcripts in situ. Science. 1998;280:585–90. 9554849 10.1126/science.280.5363.585

[23] Raj A, van den Bogaard P, Rifkin SA, van Oudenaarden A, Tyagi S. Imaging individual mRNA molecules using multiple singly labeled probes. Nat Methods. 2008;5:877–9. 18806792 10.1038/nmeth.1253 PMC3126653

[24] Liu C-W, Tian X, Hartwell HJ, Leng J, Chi L, Lu K, et al. Accurate measurement of Formaldehyde-Induced DNA–Protein Cross-Links by High-Resolution orbitrap mass spectrometry. Chem Res Toxicol. 2018;31:350–7. 29651845 10.1021/acs.chemrestox.8b00040

[25] Tayri-Wilk T, Slavin M, Zamel J, Blass A, Cohen S, Motzik A, et al. Mass spectrometry reveals the chemistry of formaldehyde cross-linking in structured proteins. Nat Commun. 2020;11:3128. 32561732 10.1038/s41467-020-16935-w PMC7305180

[26] Ongay S, Langelaar-Makkinje M, Stoop MP, Liu N, Overkleeft H, Luider TM, et al. Cleavable crosslinkers as tissue fixation reagents for proteomic analysis. ChemBioChem. 2018;19:736–43. 29356267 10.1002/cbic.201700625

[27] Tsompana M, Buck MJ. Chromatin accessibility: a window into the genome. Epigenetics Chromatin. 2014;7:33. 25473421 10.1186/1756-8935-7-33 PMC4253006

[28] Mansisidor AR, Risca VI. Chromatin accessibility: methods, mechanisms, and biological insights. Nucleus. 2022;13:236–76. 36404679 10.1080/19491034.2022.2143106 PMC9683059

[29] Hiraoka Y, Dernburg AF, Parmelee SJ, Rykowski MC, Agard DA, Sedat JW. The onset of homologous chromosome pairing during Drosophila melanogaster embryogenesis. J Cell Biol. 1993;120:591–600. 8425892 10.1083/jcb.120.3.591 PMC2119536

[30] Lu T, Ang CE, Zhuang X. Spatially resolved epigenomic profiling of single cells in complex tissues. Cell. 2022;185:4448–e446417. 36272405 10.1016/j.cell.2022.09.035 PMC9691621

[31] Takei Y, Yun J, Zheng S, Ollikainen N, Pierson N, White J, et al. Integrated Spatial genomics reveals global architecture of single nuclei. Nature. 2021;590:344–50. 33505024 10.1038/s41586-020-03126-2 PMC7878433

[32] Pandya-Jones A, Black DL. Co-transcriptional splicing of constitutive and alternative exons. RNA. 2009;15:1896–908. 19656867 10.1261/rna.1714509 PMC2743041

[33] Schwarzkopf M, Liu MC, Schulte SJ, Ives R, Husain N, Choi HMT, et al. Hybridization chain reaction enables a unified approach to multiplexed, quantitative, high-resolution immunohistochemistry and in situ hybridization. Development. 2021;148. 10.1242/dev.199847 PMC8645210 35020875

